# Integrating genomic and epidemiologic data to accelerate progress toward schistosomiasis elimination

**DOI:** 10.7554/eLife.79320

**Published:** 2022-08-30

**Authors:** Andrea J Lund, Kristen J Wade, Zachary L Nikolakis, Kathleen N Ivey, Blair W Perry, Hamish NC Pike, Sara H Paull, Yang Liu, Todd A Castoe, David D Pollock, Elizabeth J Carlton

**Affiliations:** 1 Department of Environmental and Occupational Health, Colorado School of Public Health, University of Colorado Anschutz Aurora United States; 2 Department of Biochemistry & Molecular Genetics, University of Colorado School of Medicine Aurora United States; 3 Department of Biology, University of Texas at Arlington Arlington United States; 4 Sichuan Centers for Disease Control and Prevention Chengdu China; Radboud University Medical Centre Netherlands; Radboud University Medical Centre Netherlands

**Keywords:** schistosomiasis, population genetics, surveillance, one health, disease control, whole-genome sequencing

## Abstract

The global community has adopted ambitious goals to eliminate schistosomiasis as a public health problem, and new tools are needed to achieve them. Mass drug administration programs, for example, have reduced the burden of schistosomiasis, but the identification of hotspots of persistent and reemergent transmission threaten progress toward elimination and underscore the need to couple treatment with interventions that reduce transmission. Recent advances in DNA sequencing technologies make whole-genome sequencing a valuable and increasingly feasible option for population-based studies of complex parasites such as schistosomes. Here, we focus on leveraging genomic data to tailor interventions to distinct social and ecological circumstances. We consider two priority questions that can be addressed by integrating epidemiological, ecological, and genomic information: (1) how often do non-human host species contribute to human schistosome infection? and (2) what is the importance of locally acquired versus imported infections in driving transmission at different stages of elimination? These questions address processes that can undermine control programs, especially those that rely heavily on treatment with praziquantel. Until recently, these questions were difficult to answer with sufficient precision to inform public health decision-making. We review the literature related to these questions and discuss how whole-genome approaches can identify the geographic and taxonomic sources of infection, and how such information can inform context-specific efforts that advance schistosomiasis control efforts and minimize the risk of reemergence.

## Introduction

Over the past two decades, a surge in political will and financial resources has been directed toward reducing the burden of schistosomiasis and other neglected tropical diseases (NTDs) (World Health Organization, 2012; World Health Organization, 2020; Uniting to Combat Neglected Tropical Diseases, 2022). NTDs affect approximately 1 billion people globally, largely the poor, including over 200 million people with schistosomiasis (World Health Organization, 2020). In the case of schistosomiasis, efforts have primarily focused on expanding treatment coverage through mass drug administration (MDA). MDA programs can achieve reductions in morbidity and infection intensity, but these reductions can be transient, and the outcomes have fallen short of World Health Organization (WHO) control and elimination targets (Deol et al., 2019). The disappointing reality is that across geographic regions, schistosomiasis persists despite aggressive treatment programs, and has reemerged in areas where it was previously controlled for reasons not fully understood (Wiegand et al., 2017; Kittur et al., 2019; Knopp et al., 2019; Liang et al., 2006). This has led the WHO to call for scientific advances and new tools to accelerate progress in reducing the substantial burden of schistosomiasis and other NTDs (World Health Organization, 2020).

Genomic surveillance is commonly employed as an important tool to understand the sources of viral and bacterial infection outbreaks (Köser et al., 2014; Kao et al., 2014; Quainoo et al., 2017; Pillay et al., 2020; Deurenberg et al., 2017; Popovich and Snitkin, 2017). For example, whole-genome sequencing (WGS) of clinical samples has been used to monitor drug resistance and investigate transmission clusters of *Mycobacteria tuberculosis* (Meehan et al., 2019; Satta et al., 2018). Genomic sequencing has also been used to improve surveillance of food-borne outbreaks of bacterial pathogens (Jenkins et al., 2019), to distinguish nosocomial from community-acquired *Clostridium difficile* infections (Lim et al., 2020), and to document reinfection with SARS-CoV-2 (Tillett et al., 2021). Genomic techniques are, however, still in the early stages of development for complex eukaryotic pathogens such as schistosomes. This is partly due to technical limitations caused by the greater size of eukaryotic genomes, which are more difficult and expensive to sequence than prokaryotes (Camus et al., 2002; Luo et al., 2019). Additionally, the complex reproductive life cycles of eukaryotic pathogens, including *Schistosoma*, involve both asexual and sexual reproductive phases, which requires high-resolution analytical approaches to infer patterns of relatedness and ancestry (Steinauer et al., 2010).

Advances in sequencing technologies and corresponding reductions in costs have recently made it feasible to generate whole-genome scale data on individual eukaryotic pathogens, including those that that cause schistosomiasis (Rey et al., 2021b; Nikolakis et al., 2021; Le Clec’h et al., 2018). Work to date on the genomics of schistosomes has primarily focused on developing reference genome resources for key pathogen species (Han et al., 2009; Berriman et al., 2009; Zhou et al., 2009; Young et al., 2012), which has enabled further studies of population structure, pathogen evolution, and drug response (Chevalier et al., 2014; Crellen et al., 2016; Berger et al., 2021). These advances have created new opportunities to use genomic data in population-based research and surveillance of schistosomiasis.

While the application of WGS technologies to schistosome genomics has been limited to date (Crellen et al., 2016; Berger et al., 2021; Kincaid-Smith et al., 2021; Rey et al., 2021a), recent population-level genomic studies of other eukaryotic pathogens with both sexual and asexual reproduction illustrate the potential value of integrating genomic data with epidemiological studies. Genomic data played an important role in identifying dogs as a reservoir of *Dracunculus medinensis*, the nematode that causes Guinea worm disease (Eberhard et al., 2014; Durrant et al., 2020). An investigation of the spatial scale of *D. medinensis* transmission using geocoded samples and genomic data shed light on the area that should be investigated when cases are detected (~20 km) – key information for efforts to eliminate the disease (Ribado et al., 2021). Genomic data are also being integrated into malaria surveillance and control programs to monitor drug resistance in *Plasmodium* parasites and insecticide resistance in *Anopheles* vectors (Neafsey et al., 2021) For example, surveillance for genomic signatures of resistance to artemisinin, a key malaria drug, informs regionally tailored treatment guidelines (Hupalo et al., 2016). Genomic data have been used to identify the diversity of adaptations to mosquito-control strategies, highlighting the need for whole-genome data to monitor insecticide resistance in *Anopheles* (Neafsey et al., 2021). These studies point to the broad potential for genomic data to enhance understanding of current transmission dynamics and inform infectious disease-control programming, especially in regions approaching elimination (Dalmat et al., 2019).

Similarly, population-level genomic studies of schistosomiasis can shed new light on the processes that drive schistosome infections in different social and ecological contexts, facilitating the design of tailored, region-specific control programs. Schistosome infections are acquired through dermal contact with fresh water contaminated with the larval stage of the parasite, whose life cycle requires the presence of intermediate snail hosts that live in or around freshwater bodies including laks, streams, reservoirs, and irrigation ditches. Infected humans and other mammalian hosts shed schistosomes in their urine (*Schistosoma haematobium*) or stool (*Schistosoma japonicum*, *Schistosoma mansoni*, *Schistosoma mekongi, Schistosoma intercalatum*, and *Schistosoma guineensis*). Contact rates between humans and schistosome parasites vary depending on the seasonality, composition of, and distance to aquatic habitats, the definitive host range of the pathogen species, as well as the movement patterns of humans and other mammalian hosts. Due to the aforementioned factors, the pathways driving schistosome transmission can vary regionally.

**Figure 1.**
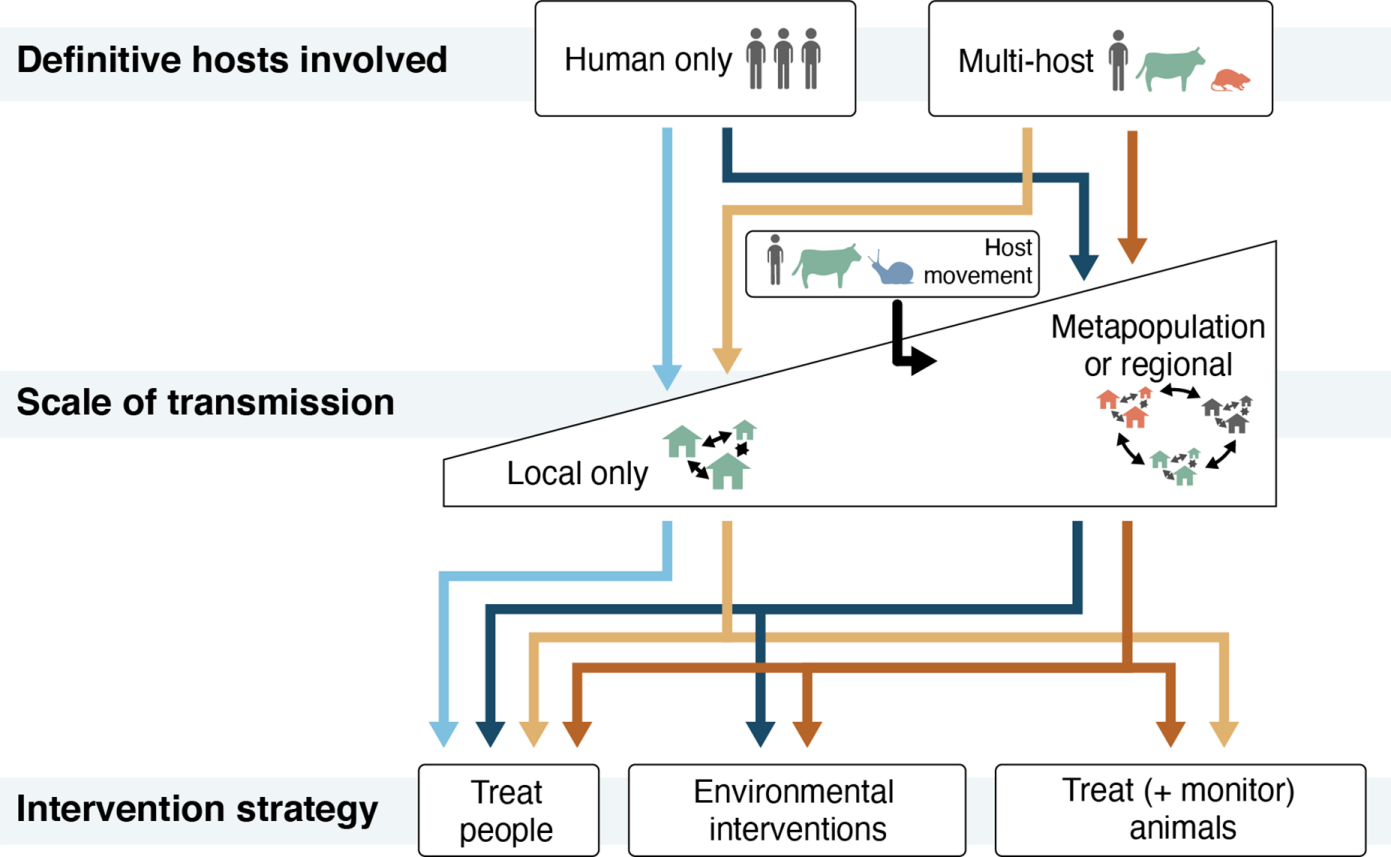
A framework for tailoring schistosomiasis control interventions to target key drivers of infection. The involvement of multiple definitive host species and the degree to which transmission occurs at local to regional geographic scales influences which interventions are most likely to be effective. Mass drug administration (MDA) is currently the primary intervention used to control schistosomiasis. We argue it is most effective at reducing the burden of schistosomiasis if infections are confined to human hosts and are acquired locally (light blue arrows). If humans are the only definitive hosts involved but human movement connects multiple transmission sites, additional environmental interventions that control transmission may be necessary to avoid post-treatment resurgence and/or re-introduction via host movement (dark blue arrows). Environmental interventions include the provision of safe sanitation to reduce the emissions of schistosomes into the environment, snail control to reduce the asexual replication of schistosomes in the environment, and the provision of abundant, cercaria-free water supplies to reduce exposure through water contact. If multiple definitive host species are involved in transmission, veterinary interventions that target non-human hosts (e.g., livestock treatment or surveillance of wildlife populations) should be layered onto MDA if infections are acquired locally (yellow arrows) or implemented in combination with MDA and environmental interventions if infections are acquired across regional scales (red arrows).

We argue that accelerating progress in control of schistosomiasis requires the ability to define context-specific drivers of infections in ways that can be effectively translated to intervention strategies. Two interrelated questions are key to inform the design of interventions tailored to distinct eco-epidemiological circumstances: (1) how often do non-human host species contribute to human infection? and (2) what is the importance of locally acquired versus imported infections in driving transmission at different stages of elimination? (Figure 1). As we discuss below, population genomic-scale approaches integrated with epidemiological and ecological studies provide high-resolution answers to these questions, offering a level of precision that is actionable from a public health perspective, such that schistosomiasis-control programming can be tailored to region-specific contexts.

In this review, we outline how whole-genome sequence data can be embedded into research and surveillance of schistosomes to bridge gaps in our understanding of schistosomiasis and to advance progress in control and elimination efforts. We provide an overview of historical, current, and emerging genomic approaches for studying schistosomiasis, including a critical review of key studies over the last two decades. Looking forward, we describe the advances that make it increasingly feasible to integrate genomic data into epidemiological studies of schistosomiasis. We then summarize two decades of research on schistosome epidemiology, ecology, and population genetics relevant to our priority questions, highlighting what is known and what knowledge gaps remain, and how the integration of genome-scale information can help fill these gaps. We consider how intervention strategies can be driven by high-resolution genomic inferences about the extent of non-human mammalian host involvement and the importance of imported infections in driving local transmission. We also consider how the relative importance of pathogen importation and different mammalian hosts may shift over time due to the success of control programs and selective pressure on the schistosome genome, and discuss how genomic surveillance can provide early warnings of biologically relevant shifts. We conclude by discussing key considerations and challenges for applying genomic data to schistosomiasis surveillance, including the trade-offs between cost and sampling breadth with respect to the use of genomic data in population-based studies, and the ongoing efforts to build capacity for the generation and analysis of genomic data in schistosomiasis-endemic countries. Throughout we aim to show how including genomic approaches in schistosomiasis research and surveillance can enable strategic data-driven interventions that effectively address local infection and transmission contexts.

## A brief history of genetic methods in schistosomiasis epidemiology

To catalog how genetic data have been used to inform our understanding of human schistosomiasis to date, we critically reviewed a representative, non-exhaustive set of 47 studies that have used molecular methods to study human schistosomiasis since 2005 (search terms are provided in Appendix 1). These studies are compared in terms of the research questions investigated, spatial scale, and data generation methods (Table 1, summary of full list of studies provided in Supplementary file 1). In doing so, we update a 12-year-old foundational review on the topic (Steinauer et al., 2010) and build on recent work highlighting how population genetics can shed light on schistosome evolution and adaptation (Rey et al., 2021b). Below, we review the early challenges in generating genetic data, the widespread adoption of microsatellite (MSAT) loci for genetic studies, and the more recent application of newer sequencing technologies. We provide a primer describing the approaches that have been used over the past two decades, along with their strengths and weaknesses, in Box 1.

Box 1. **Molecular techniques used to study genetic and genomic variation in schistosomes.**

*Microsatellites (MSATs)*. MSATs are short tandemly duplicated sequences, made up of repeated copies of 2–6 bp, predominantly found in non-coding regions of the genome. MSATs can accumulate copy repeat mutations rapidly, making them highly variable within a population and useful to assess population divergence and connectivity (Fischer et al., 2017; Väli et al., 2008). Limitations of MSATs include difficulty in replicating allele scoring across laboratories (Morin et al., 2009), constraints on the number of loci that can be scored per individual (often limited to a few dozen loci), and the need for species-specific primers to amplify loci (Chapuis and Estoup, 2007). Because they reflect non-coding regions, MSATs are not appropriate for addressing natural selection and more ancient processes of introgression (Kim et al., 2007), and homoplasy (repeated mutation to the same allele) can decrease the inference accuracy (Hansson and Westerberg, 2002).

*Mitochondrial DNA (mtDNA) and nuclear ribosomal DNA (rDNA) sequencing*. The mtDNA in eukaryotic cells is maternally inherited and codes for proteins and RNAs that are important for mitochondrial function and cellular metabolism. rDNA genes encode eukaryotic ribosomal RNAs, which comprise critical functional components of cellular ribosomes. Both rDNA and mtDNA tend to occur in high copy number, making them easy to amplify and sequence, and they have been used to infer broad-scale patterns of relatedness among schistosome populations and species (Catalano et al., 2020; Boon et al., 2018; Huyse et al., 2009). However, variation in these markers is limited: they provide only course-scale inferences of relatedness that are often insufficient in resolution to address questions about transmission or host contributions at fine scales, and they have limited power to detect and to characterize hybridization between species.

*Reduced representation (RR) sequencing*. There are many forms of RR sequencing, but the most common approaches include RADseq (Altshuler et al., 2000) and ddRADseq (Peterson et al., 2012), which use restriction enzymes to cut the genome at predictable locations and then sequence these fragments. This results in thousands of sequenced loci shared (and thus comparable) among individual parasites with minimal up-front protocol customization. RR leverages the power and economy of modern sequencing technologies, and is scalable to infer fine-scale population structure and broad-scale estimates of relatedness (Shortt et al., 2017; Shortt et al., 2021; Lozier, 2014). However, there may be lineage-specific dropout and high variance in sequencing coverage across loci and among laboratories, resulting in sparse data matrices that reduce replicability and lower precision (Lowry et al., 2017).

*Exon capture sequencing.* Exon capture sequencing uses biotinylated oligonucleotide probes designed to target exonic sequences from a reference genome, thus focusing sequencing efforts on exon-encoding regions that may be linked to parasite phenotypes of interest (e.g., drug resistance and host specificity), and reducing the amount of data needed while still providing a genome-wide perspective on variation (e.g., Le Clec’h et al., 2018). Exon sequence capture produces more even coverage than RR approaches, provides information on functionally relevant protein-coding regions and has been used to identify regions that introgressed between schistosome species in samples from across the African continent (Platt et al., 2019). Limitations of exon capture include the need for high-quality reference genomes for species-specific probe design, costs associated with exon-targeted probes, and the exclusion of cis-regulatory sequences that may control gene expression.

*Whole-genome sequencing (WGS).* Sequencing the entire nuclear genome allows for precise estimates of genetic diversity, relatedness, and selection compared to other methods (Allendorf et al., 2010; Bradbury et al., 2015). Sample preparation and data collection are standardized, making it replicable across laboratories (Krawczak, 1999; Nielsen, 2000) and facilitating comparisons across samples and among studies. WGS includes all genomic regions that may be relevant to characterize responses to control measures, host selection, and transmission dynamics (Väli et al., 2008; Brumfield et al., 2003; Pool et al., 2010). The primary drawback of WGS is the high per sample sequencing cost, which may limit the number of individuals sampled and encourage low-coverage sequencing, but ongoing improvements in technology and reduced costs favor its use in the future.

**Table 1.** Number of studies of schistosomiasis epidemiology employing molecular methods by data generation method, study question, and study scale.

	Data generation method												
	Microsatellite				mtDNA/rDNA				RAD/Exome/WGS				
**Study question**	*LAB*	*LOC*	*REG*	*CON*	*LAB*	*LOC*	*REG*	*CON*	*LAB*	*LOC*	*REG*	*CON*	**Total**
Population structure	0	6	2	2	0	1	0	2	0	2	0	0	15
Hybridization	0	1	1	0	0	5	1	0	0	0	0	1	9
Drug resistance	0	5	0	0	0	1	0	0	1	2	0	0	9
Host species identification	0	5	0	0	0	3	0	0	0	0	0	0	8
Lineage origins/diversification	0	0	0	1	0	0	0	1	0	0	1	3	6
Morbidity/phenotype	0	3	0	0	0	0	0	0	0	0	0	0	3
Transmission persistence	0	1	0	0	0	0	0	0	0	0	0	0	1
Worm natural history	0	0	0	0	0	0	0	1	0	0	0	0	1
**Total**	0	21	3	3	0	10	1	4	1	4	1	4	47*

Study scale abbreviations: LAB = laboratory study; LOC = local scale (tens of km; neighboring villages); REG = regional scale (hundreds of km; neighboring countries); CON = continental scale (thousands of km; non-neighboring countries). Categories informed by those used by Rey et al., 2021a; *Adding totals across question types and generation methods exceeds 47 because some studies employed multiple data generation methods (e.g., they use both microsatellites and mtDNA markers) and/or addressed multiple types of questions (e.g., population structure and host species identification). Search terms used to identify these studies are provided in Appendix 1. A list of all studies included in this table is provided in Supplementary file 1 and another version of this table that lists which studies are counted in which cells is provided in Supplementary file 2.

### The challenge of DNA quantity

A key historical challenge in generating population genomic data from schistosomes is the limited DNA quantity from readily available life stages. Infected humans and other definitive hosts shed eggs, which hatch into miracidia, while infected snails shed cercariae. Both miracidia and cercariae can be archived on FTA cards (cotton-based, cellulose paper cards containing chemicals that lyse cells, denature proteins, and preserve DNA) under basic laboratory conditions, allowing sample collection from both definitive and intermediate hosts in field settings. However, miracidia and cercaria contain only a few nanograms of DNA. In contrast, adult schistosomes contain larger quantities of DNA but live inside definitive hosts and can only be obtained through autopsy, thereby limiting their utility for population-level studies. A notable exception is opportunistic worm collection from domestic animals at the time of slaughter (Jiz et al., 2021). Early efforts to generate genetic data from field-collected schistosome samples required laboratory passage of these parasites to extract DNA from adult worms harvested from laboratory animals (Steinauer et al., 2010). Laboratory passage added weeks to the process and was prone to population bottlenecks (Shrivastava et al., 2005; Gower et al., 2007).

Early studies of schistosome larvae confronted the issue of limited DNA quantity by sampling a small number of loci using single and then multi-plex PCR assays (Steinauer et al., 2010). These early studies relied on a limited number of microstatellite loci (5–20 among the studies reviewed in Supplementary file 1 ). A breakthrough for large-scale genomic data generation came when whole-genome amplification (WGA) methods were applied to larval schistosomes, with early examples analyzing over 50 MSATs at high fidelity (Valentim et al., 2009) or a somewhat more limited panel (Xiao et al., 2013). Over the past 5 years, WGA has been used to generate genome-scale data from individual schistosomes representing all three major human schistosome species (Nikolakis et al., 2021; Le Clec’h et al., 2018; Shortt et al., 2017; Shortt et al., 2021). WGA of larval schistosomes and other species yields low error rates with minimal genome-wide amplification biases (Nikolakis et al., 2021; Shortt et al., 2017; Valentim et al., 2009; Blair et al., 2015), making WGA a suitable method for generating genomic data from larval schistosomes, including miracidia samples preserved on FTA cards and stored for up to a decade or more prior to sequencing (Le Clec’h et al., 2018; Shortt et al., 2017).

### MSAT markers

As mentioned above, MSATs provided some of the first inferences of fine-scale patterns of relatedness among schistosomes (Shrivastava et al., 2005; Wang et al., 2006). They were widely adopted: most studies we reviewed (27/47) employed MSATs. These studies addressed a diversity of questions related to parasite population structure (10/27), host species identification (5/27), and drug resistance (5/27) primarily at local scales (where data collection occurred in neighboring villages tens of kilometers apart, 20/27 studies) (Table 1). Applications include inferences of approximate relatedness among miracidia [e.g., Gower et al., 2017], population structure across landscapes (Gower et al., 2013; Rudge et al., 2009; Barbosa et al., 2013; Prugnolle et al., 2005), and the impacts of drug treatment on parasite genetic diversity (Blanton et al., 2011; Norton et al., 2010). MSATs can be used to infer approximate patterns of relatedness between parasites and among parasite populations (Box 1). However, because MSATs involve relatively few idiosyncratic and unmapped loci, they are unable to shed light on genetic variants that may be implicated in driving adaptive change and limited numbers of MSAT loci provide less precision for inferring levels and patterns of kinship than tens of thousands of genomic loci. Moreover, allele scoring can be difficult to replicate across laboratories, raising questions about the suitability of this approach for widespread surveillance. It should be noted that MSATs may remain cost effective, or the only option, when laboratories are already heavily invested in MSATs, or when labor costs are low compared to the costs of accessing sequencing technology.

### mtDNA and nuclear rDNA sequencing

About one-third of studies we reviewed (15/47) used mitochondrial DNA (mtDNA)/ribosomal DNA (rDNA) markers, primarily at local (10/15) and in some cases continental (4/15) scales (where samples are derived from sites in non-neighboring countries located thousands of kilometers apart; Table 1). These studies most frequently addressed questions of hybridization between schistosome species (5/15) and have also been used to identify host species (3/15). The abundance of mtDNA and rDNA copies per cell makes these markers easy to amplify and sequence, but relatively low levels of variation and the maternal inheritance for mtDNA limit the ability to use these markers to infer fine-scale relatedness (Box 1). Due to the highly conserved nature of these regions, these methods can be used to examine broad-scale patterns of relatedness, and for initial inferences that hybridization may be occurring. However, due to the reliance on a single locus, they provide limited power and resolution to detect hybridization, yielding a high false-negative rate and a non-zero false-positive rate. They are not well suited to studies focused on the genomic impacts of control efforts, host compatibility, and population differentiation, and for inferring fine-scale patterns of relatedness (Box 1).

### Genome-scale approaches

Genome-scale approaches (i.e., reduced representation [RR], exome capture or full-scale WGS; described in detail in Box 1) have only recently become feasible; all studies in our review that used these approaches were published in 2014 or later. Such studies are becoming increasingly common due to their increasing cost efficiency in obtaining data and we anticipate that the number of studies using these approaches will grow in the years ahead. Only 10 of 47 studies we reviewed used genome-scale approaches. These studies have been applied to a broad range of questions including about the origins, divergence, and differentiation of lineages (4/10) and drug resistance (3/10) and were carried out at both local (4/10) and continental (4/10) scales (Table 1). The diverse nature of studies reflects the relatively recent emergence of these technologies, their broad potential applications and the reductions, even over the past decade, in the costs associated with these approaches. For example, an early study used WGS approaches to examine 10 schistosomes on a continental scale to evaluate the divergence of two schistosome species (Crellen et al., 2016). More recently, genome-scale approaches have been used to examine hundreds of miracidia to infer patterns of infection at fine spatial scales (e.g., Shortt et al., 2021; Berger et al., 2021; Vianney et al., 2021). WGS, in particular, has become an increasingly compelling option for population-based studies, the reasons for which we outline in the next section.

### The new frontier: WGS in schistosome populations

Advances in reference genomes, together with WGA, have facilitated the use of sequencing for population-level studies of schistosomes. Over the past decade, reference genomes became available for the three species of *Schistosoma* that cause most cases of human schistosomiasis: *S. mansoni, S. haematobium*, and *S. japonicum* (Han et al., 2009; Berriman et al., 2009; Zhou et al., 2009; Young et al., 2012). By providing a basis for understanding schistosome genes and genetic variation, these annotated reference genomes contributed to the identification of the genomic basis of key host-parasite interactions, including genes tied to utilization of host nutrients for parasite growth and development (Zhou et al., 2009), and have extended our understanding of molecular pathways involved in schistosome life histories (Berriman et al., 2009). These genome resources also provided the foundations for further studies that identified schistosome genes and genetic variants linked to drug resistance and susceptibility (Le Clec’h et al., 2021) and host preference (Luo et al., 2022), and provided insights into the genomic impacts of control measures (Berger et al., 2021). Schistosome reference genomes have been improved and updated, and all three are now high-quality chromosome-level reference genome assemblies (Wit and Gilleard, 2017; Xu et al., 2014).

Genome sequencing data can come in the form of RR techniques, exon sequencing, or WGS. Despite the greater per sample costs of sequencing, it produces larger amounts and more informative data for the cost and opens new opportunities to address questions related to the epidemiology, treatment, and control of schistosomes using high-resolution data that were otherwise either difficult or not possible to address with prior genetic approaches. Studies using WGS have estimated precise divergence times between *S. mansoni* and *S. rodhaini* (Crellen et al., 2016), demonstrated the genome-wide impact of repeated MDA on populations of *S. mansoni* with documented reductions in praziquantel efficacy (Berger et al., 2021), and resolved fine-scale population structure and relatedness of *S. japonicum* infections within and between villages (Nikolakis et al., 2021; Shortt et al., 2021). By obtaining genetic variation from across the entire genome, WGS greatly improves the precision of estimates of genetic diversity, relatedness, and population structure compared to other methods (Nikolakis et al., 2021). Genomic methods such as RR and exon sequencing (see Box 1), which sequence only a fraction of the genome, often a few percent of all bases, can provide fine-scale population structure information. WGS can provide even higher resolution and better pedigree information, while also providing the opportunity to identify patterns of variation in genes and functionally relevant genomic regions that can shed light on parasite evolution, including rapid evolution in response to control measures.

High-resolution estimates of genetic variation across entire genomes are particularly important for studying schistosomes, whose combination of sexual and asexual reproduction complicates inferences of relatedness. These biological characteristics can lead to a high degree of inbreeding that can be further exacerbated by population bottlenecks when control efforts reduce parasite population sizes (Blanton et al., 2011; Norton et al., 2010), though control efforts may not always lead to such bottlenecks (Faust et al., 2019). Importantly, high-resolution genomic approaches can be used to precisely and accurately infer the relatedness of parasite samples, informing transmission dynamics at fine geographic and temporal scales. This fine-scale resolution of parasite relatedness (e.g., confidently resolving sibling and other close-order relationships in the context of potentially highly inbred populations) (Shortt et al., 2021), and its importance for inferring transmission events and patterns, represent key advancements to help understand the sources of new infections. These advancements can help identify which transmission pathways drive schistosome infections in a given region, including the contributions of non-human definitive hosts and parasite import (Figure 1). In the sections below, we describe what is known about the two questions we articulated in the introduction, including how genetic and genomic data have contributed to our understanding, and discuss how the use of genome sequencing can help fill remaining gaps.

## Priority Question 1: How often do non-human host species contribute to human schistosomiasis?

Until recently, concern about which mammalian species may contribute to the transmission of schistosomiasis focused on *S. japonicum* in Asia, a known zoonosis with over 40 wild and domesticated animal species identified as competent reservoirs (Liu et al., 2016). By contrast, the contribution of non-human mammals to the transmission of the human schistosomes *S. mansoni* and *S. haematobium* was considered negligible (Bustinduy and King, 2014). However, this assumption was challenged by recent work in West Africa that has demonstrated the potential for other mammalian hosts to play a role in transmission cycles when schistosomes use animals as non-human mammalian reservoirs or through the hybridization of human and animal schistosome species (Boon et al., 2018; Catalano et al., 2018). As control progresses in human populations, even infrequent animal involvement has the potential to perpetuate the transmission cycle and impede elimination targets (Colley and Loker, 2018). Moreover, as we discuss below, evolutionary pressures, particularly in later stages of control efforts, may lead to new adaptations such as shifts in host range that circumvent constraints imposed by control measures.

As we highlighted in Figure 1, when non-human reservoirs serve as sources of human schistosome infections, schistosomiasis control strategies should address that route of infection. Below, we review a diverse evidence base, including epidemiological and ecological studies, mathematical modeling simulations, and studies using genetic and genomic approaches, to evaluate the role of non-human definitive hosts in the transmission of the three major human schistosomes. The evidence indicates that the contributions of non-human mammalian hosts are context specific, and the extent of the involvement of various potential hosts and their relevance to control may shift over time. We demonstrate how high-resolution genomic approaches, when paired with epidemiological and ecological assessments, can be used to discern the extent to which zoonotic hosts contribute to human schistosomiasis in areas of persistent, reemerging, or newly emerged schistosomiasis and to resolve current gaps in the scientific knowledge.

### Zoonotic contributions to *S. japonicum* infections vary regionally

There is robust evidence that bovines (cattle and water buffalo) are key contributors to human schistosomiasis in the lake and marshland regions of China, one of the most well-studied regions in the world regarding the role that non-human hosts play in schistosome transmission. Interventions targeting treatment to bovines (Gray et al., 2009) and policies to replace bovines with agricultural machinery (Liu et al., 2017) led to declines in human schistosomiasis in this region, strong evidence that bovines contribute to human infection risk, and justification of interventions to treat bovine populations (Figure 1). In addition to these experimental studies, mathematical modeling (Rudge et al., 2013; Williams et al., 2002) and early genetic studies using MSAT loci (Rudge et al., 2009; Lu et al., 2010a) similarly pointed to a central role of bovines in human schistosomiasis. There is strong biological plausibility for these conclusions: in this region, the use of bovines in farming leads to the overlap of human and bovine activity with snail habitat. Moreover, bovines are estimated to deposit up to 90% of the *S. japonicum* eggs in this area due to their high densities, voluminous fecal output, and high infection intensity (Guo et al., 2001).

However, outside of this region, the role of bovines and other mammalian hosts in human schistosomiasis japonicum is less clear. Studies using relatively limited numbers of MSAT loci to estimate gene flow and relatedness between schistosomes from definitive and intermediate hosts in Anhui, China, and in the Philippines found evidence of high levels of gene flow between humans and other mammalian hosts sampled including rodents, dogs, and, in the case of the Philippines, pigs and water buffalo (Rudge et al., 2009; Lu et al., 2010a; Rudge et al., 2008). At face value, these studies suggest a transmission cycle involving multiple mammalian hosts. However, the conclusions from these studies are limited by the low resolution of the small numbers of MSAT loci used, such that it is difficult to confidently discern whether evidence of gene flow and relatedness reflects a true multi-definitive host transmission cycle, or if the appearance of such transmission is a spurious conclusion. In other words, had a high-resolution approach been used, would a more limited group of definitive hosts be implicated in human infections? It is biologically plausible that the role of different host species varies by context: population density, infection intensity, and average stool output of different host species have been hypothesized to explain the contribution of zoonotic hosts to human *S. japonicum* infections (Wang et al., 2005).

High-resolution genomic approaches, paired with epidemiological and ecological assessments, can be used to discern the extent of zoonotic host involvement in human schistosomiasis japonicum, providing precise estimates of parasite relatedness. Specifically, genome-scale approaches can be used to identify the number of between-species (non-human to snail to human) transmission events, the geographic origins and scale of such events, and the degree to which they have occurred in the past or are ongoing. Such approaches can also provide accurate inferences of recent human to snail-to-human transmission events to reduce the inferred probability of non-human host involvement in new infections (Shortt et al., 2021). The use of methods such as WGS opens the opportunity to evaluate the relative contributions of different host species to the human transmission cycle in distinct regions, and evaluate candidate metrics for estimating host involvement (e.g., as a function of density, stool output, and infection intensity).

It is also plausible that the importance of non-human hosts shifts over time. For example, *S. japonicum* infections in humans and bovines have declined in China since the 1980s, but infections in rodents have remained relatively stable (Zou et al., 2020). This suggests that rodents may become a more important source of schistosome infection as the region approaches elimination targets and highlights an ongoing need for genomic surveillance of schistosomes among rodent populations to inform control programs in China and in other areas and host species.

We note that a key first step in assessing definitive host involvement is testing non-human hosts for *S. japonicum* infection and collecting schistosomes from infected hosts. Assessing infection prevalence or intensity in different species can approximate potential host involvement (e.g., no infections in a species is strong evidence that the species is not contributing to transmission) (Lu et al., 2010b). However, accurate assessment of infections by species can be challenging, particularly in low-prevalence settings where infrequent infections and poor diagnostic performance can fail to detect parasite populations (Liang et al., 2022) and, thus, bias assessments of species involvement in human schistosomiasis (Wu et al., 2010). Guidance on best practices for surveillance of schistosome infections in non-human hosts is an important priority that will improve the rigor of studies of non-human host involvement.

### Using genomic epidemiology to detect shifts in *S. mansoni* host specificity

The availability of non-human hosts in different contexts may influence the host specificity for *S. mansoni* in similar ways as for *S. japonicum*. Rodents are competent hosts of *S. mansoni*, though schistosomiasis mansoni is considered a predominately human disease. Prior to the availability of genetic approaches, the involvement of rodent hosts in human cases of *S. mansoni* was assessed by studying the chronobiology of the parasites’ cercarial emergence from snails, which is adapted to the timing of water contact behavior in the primary definitive host for each species in a given habitat (Combes et al., 1994). For example, *S. mansoni* on the island of Guadeloupe occurred as three chronotypes that reflected the availability of hosts in different habitats: (1) in urbanized areas where humans were the primary host, cercariae emerged early in the day, (2) in sylvatic areas with primarily rodent hosts, cercariae emerged late in the day, and (3) in mixed foci of infection where both humans and rodents were available, the peak in cercarial emergence was bimodal (Theron and Pointier, 1995). Cercarial shedding patterns of *S. japonicum* have also been found to vary with the water contact patterns of the dominant hosts in an area (late afternoon in rodent-dominant areas and morning in bovine-dominant areas; Lu et al., 2009), indicating that variation in chronobiological traits is likely a common phenomenon across schistosome species.

More recent genomic approaches have been incorporated into epidemiological and ecological studies of the *S. mansoni* host range, and these studies have demonstrated the potential importance of non-human hosts in human *S. mansoni*. One recent study detected *S. mansoni* infections in small mammals in West Africa using mtDNA/rDNA markers (Catalano et al., 2020) and another using MSATs found little genetic differentiation among *S. mansoni* infecting humans and non-human primates in Ethiopia (Kebede et al., 2020). These studies suggest that, at least in some contexts, the *S. mansoni* transmission cycle includes both humans and other definitive hosts. Two high-resolution genomic approaches can further resolve the participation of other mammals in human *S. mansoni* infections. As in the case of *S. japonicum*, using WGS to estimate fine-scale relatedness of parasites collected from humans and other mammalian hosts can provide robust evidence whether non-human mammalian hosts participate in a shared transmission cycle with human hosts in a given region. Second, identifying and monitoring genetic variation in parasite populations associated with chronotype variation and shifts in host use are also possible with WGS. The current evidence suggests that these chronobiological patterns are adaptive and genetically determined (Théron, 2015). If the host specificity of *S. mansoni* can shift in response to selective pressure, control efforts that target human hosts without surveillance in other competent definitive host species may fail to achieve elimination targets. Genomic surveillance in animal populations and surveillance for genetic signatures of chronobiological shifts may provide early warning signs of shifts away from host-specific transmission and the need to target animal populations in control programs (Figure 1).

### Hybridization adds genetic diversity to *S. haematobium* and can complicate control efforts

Hybridization between schistosome species, particularly those within the *S. haematobium* complex, complicates schistosome host specificity and may vary across geographic contexts (Leger and Webster, 2017). A more precise understanding of such processes has the potential to inform control efforts. Hybrids occur when parasites from different species pair within the same definitive host and produce eggs that may be viable, thereby resulting in hybrid individuals that contain mixtures of genetic variants from multiple species. Hybridization can introduce new genetic variation into parasite populations and may, in turn, affect epidemiologically relevant traits, such as chronobiology, host range, and drug efficacy (Leger and Webster, 2017). Indeed, the detection of hybrids, particularly between *S. haematobium* and *S. bovis*, complicates the assumption that urogenital schistosomiasis caused by *S. haematobium* remains an exclusively human disease. Monitoring of parasite populations for evidence of hybridization can identify the presence and scope of this phenomenon, and identify potential non-human sources of infection that may otherwise undermine treatment-based programs.

Prior studies found evidence of schistosome hybridization across a diversity of settings in West Africa using mtDNA/rDNA markers (Huyse et al., 2009; Webster et al., 2013; Webster et al., 2020). *S. haematobium × S. bovis* hybrid schistosomes were found to infect rodents in Senegal (Catalano et al., 2018) and cattle in Benin (Savassi et al., 2020), though in small numbers, showing that schistosome hybrids occur naturally and are viable. It is unclear from these studies, however, whether genetic evidence of hybridization between *S. haematobium* and *S. bovis* is an ongoing phenomenon or an artifact of ancient introgression events, as such distinctions are challenging to resolve without high-resolution genomic data. As described above, the sex-biased and linked inheritance of mitochondria and the limited variation in mtDNA/rDNA can allow detection of hybridization, though with a considerable risk of false negatives. Such approaches are also limited in their ability to resolve historical dynamics or examine the biological consequences of hybridization.

In contrast, whole-genome approaches can increase the capacity of researchers to (1) distinguish ongoing hybridization from historical introgression events, (2) characterize the impacts of hybridization on the entire genome and, consequently, schistosome biology, and (3) infer the timing and ongoing nature of hybridization (Rey et al., 2021b). Indeed, recent genome-scale analyses of hybridization demonstrated that introgression is widespread in some regions with gene flow from *S. bovis* to *S. haematobium* (Rey et al., 2021a; Platt et al., 2019). Priorities within the *S. haematobium* complex include identifying geographic regions with evidence of hybridization or introgression, and understanding the degree to which hybridization is ongoing. Genome-scale approaches in geographic regions with evidence of hybridization can thus distinguish whether hybridization events are frequent or occasional sources of gene flow, whether zoonotic reservoirs are important or minor sources of hybrid infections in humans, as well as whether hybridization may alter the chronobiology of cercarial emergence and host range. Greater understanding of hybridization among schistosomes represents an important priority for understanding which definitive host species to target, where to target non-human definitive hosts, and whether additional shifts in parasite biology need to be considered.

## Priority Question 2: What is the importance of locally acquired versus imported infections?

Another key area where high-resolution genomics approaches can provide valuable insights is in addressing whether schistosome infections are being acquired locally or are imported. Importation, which we define as the introduction of schistosomes from outside a catchment of control effort, may occur as an isolated event or a regular flow of parasites. Schistosomes may be imported by the movement of infected humans, infected non-human mammals, or the dispersal of infected snails, miracidia, or cercariae through hydrological networks. The importation of schistosomes can lead to the establishment of new transmission foci in naïve populations living in environments with suitable conditions for transmission (e.g., Boissier et al., 2016; Oleaga et al., 2019). Importation may also maintain transmission in endemic areas, compromising locally targeted control programs (Gurarie and Seto, 2009; Perez-Saez et al., 2015). Even rare occurrences of parasite importation into suitable habitat could undermine elimination targets if a few founding schistosomes rapidly expand to establish a new population. In this section, we review what is known about schistosome importation, including the relative importance of local versus imported infections and the pathways of importation. We describe the how genomic approaches can be used with epidemiological methods to assess the frequency of importation events, identify their origins, determine what types of host movement matter, and characterize recent population bottlenecks and expansions. This information can help to determine whether environmental interventions are warranted, and if so, at what scale (Figure 1).

The importation of schistosomes has been responsible for the establishment of new foci of transmission in a variety of geographic settings. Population genetic and genomic data has played an important role in identifying probable importation routes for these new foci and was a key tool used to investigate a recent outbreak. The investigation of the 2014 outbreak of autochthonous schistosomiasis in Corsica, France, incorporated genomic methods, first to identify the infecting schistosome species (*S. haematobium*, *S. bovis*, and hybrids of the two species) and then to detect the likely source of imported schistosomes (human movement from West Africa) (Boissier et al., 2016; Berry et al., 2014). Similarly, the introduction of *S. mansoni* to northern Senegal after dam construction in the 1980s was also attributed to the arrival of agricultural workers from other parts of West Africa using phylogeographic analysis of mtDNA sequences (Van den Broeck et al., 2015). Perhaps the most consequential case of schistosome importation is the introduction of *S. mansoni* to the western hemisphere. This was long hypothesized to be related to movement of people through the Trans-Atlantic Slave Trade, and is now corroborated by whole-genome evidence (Crellen et al., 2016). These examples provide a template for future investigations of novel foci of transmission, which may become more common as climate change continues to alter intermediate host range (De Leo et al., 2020).

In endemic areas, the importation of schistosome parasites may help sustain transmission and contribute to reemergence following the implementation of control programs. Importation events may also represent sources of new parasite genomic variation that may influence biologically and epidemiologically relevant traits in local parasite populations. Modeling simulations suggest that both social and hydrological connectivity can sustain transmission, even in regions that would not support ongoing transmission in the absence of importation (Gurarie and Seto, 2009), that human movement may delay the interruption of transmission (Perez-Saez et al., 2015), and that infection risk is strongly influenced by snail dispersal (Ciddio et al., 2017). Models parameterized with field data from Sichuan, China, were consistent with these conclusions, indicating that most villages received parasite inputs from outside the village, either through hydrological or social connectivity (Spear, 2012). However, a study that used genomic approaches (ddRADseq) in the same region demonstrated that transmission tends to be local (occurring primarily within villages with relative temporal stability), with occasional importation of cases whose impact on transmission is not well understood (Shortt et al., 2017). A review of genetic studies across the African continent similarly estimated that gene flow among schistosome populations was confined to local scales (Rey et al., 2021b). The distinct conclusions of the population genomic and modeling analyses described above highlight the importance of genomic approaches as they can resolve the extent to which importation is contributing to local transmission in a given context. The use of genomic approaches to monitor the relative importance of parasite importation may justify a phased approach to schistosomiasis control, focusing on local sources of infection when they predominate and transitioning to a control strategy that addresses potential geographic sources of infection when and if importation is shown to contribute meaningfully to local infections, as may be anticipated when approaching elimination targets.

There are two plausible routes of parasite importation: movement of the snail and larval schistosomes via hydrological networks, and movement of definitive hosts. Empirical studies on snail dispersal use a variety of ecological and population genomic methods in diverse geographic settings and have demonstrated the plausibility of parasite importation through hydrological transport of intermediate hosts. Genotyping of snails from all three genera that transmit human schistosomes (*Bulinus*, *Biomphalaria* and *Oncomelania* in Egypt, East Africa, and China, respectively) using MSATs revealed gene flow among snail populations and indicated that snail movement can introduce schistosomes from other villages kilometers away (Zein-Eddine et al., 2017; Standley et al., 2014; Head et al., 2016). Using remotely sensed data, *Bulinus* snails with a strong propensity for aestivation were also found to readily disperse from ephemeral habitats in coastal Kenya, even during a drought (Clennon et al., 2007). Ecological field studies in Sichuan, China, indicated that both *S. japonicum* cercariae and *Oncomelania hupensis* snails are capable of dispersing through irrigation networks and reaching new sites or villages (Lowe et al., 2005; Akullian et al., 2012). In contrast to Sichuan-based field studies, analysis of MSATs from snail populations in the middle and lower reaches of the Yangtze River indicated relatively low levels of gene flow among snail populations (Guan et al., 2016). Together, these studies demonstrate the possibility of importation via hydrological transport of snails, but further investigation of how often and under what circumstances this occurs is warranted for effectively targeting – both spatially and temporally – environmental interventions to mitigate the public health impact of importation events.

The movement of infected people and animals is another potentially important source of imported schistosomes. While the modeling studies mentioned above indicate that human population movement can maintain transmission in endemic areas, the results of empirical studies highlight the gaps in our understanding of which types of human movement matter for the spread of schistosomiasis. A genetic study using MSAT markers compared parasite diversity among urban migrants and urban-born people in Brazil and inferred that migration contributed little to cases of urban schistosomiasis (Blanton et al., 2015). An epidemiological study found rural residents in Sichuan, China, were less likely to be infected with *S. japonicum* if they engaged in circular labor migration to urban areas, suggesting travel (and decreased time spent in endemic villages) is protective against infection (Buchwald et al., 2021). In contrast, migrants to three villages in Hunan province accounted for over half of all observed *S. japonicum* infections (Cao et al., 2015). This evidence highlights how diverse human movement patterns can be: whether origins and destinations are rural or urban influence if parasites are transported by host movement and if ecological conditions in a destination can even support transmission. Even less is known about the role that non-human mammalian hosts may play in the transport of schistosomes across landscapes.

To understand the role of human and other definitive host population movement in schistosome importation, we need to identify the types of movement that are most epidemiologically relevant. The ecological requirements of the parasites mean that, with few exceptions, human movement between rural areas is arguably most relevant to schistosome transmission. However, movement between rural areas is poorly accounted for in popular modeling frameworks that are well suited to capture urban migration and commuting patterns (Meredith et al., 2021; Wesolowski et al., 2015). An additional challenge is the disconnect between human and pathogen movement: a person’s potential exposure to or emission of schistosomes can seldom be discerned from their movement data alone, making it difficult to determine which movements are epidemiologically meaningful. This is a key area for the contribution of genome-scale data. Integration of genomic data from parasites with candidate measures of host movement would provide the means to evaluate the importance of different types of movement in the importation of infections as has been done with malaria (Wesolowski et al., 2018). The high resolution of genomic approaches can also reveal source-sink scenarios between hosts (human or otherwise) and geographic locations.

We suspect the risk of importation depends on context, ranging from the hydrological environment through which snails disperse to the prevailing patterns of human and animal movement. Because population genomic analyses previously indicated that infections are primarily acquired locally, it is crucial to characterize how often importation events occur and whether the importance of social and environmental connectivity changes over time, either with seasons or as regions approach elimination targets. Indeed, genomic data, coupled with human and ecological data, has an important role to play in quantifying how often importation occurs and which types of movement by which hosts drive importation events. For elimination programs to be successful, control efforts should consider the spatial extent of parasite gene flow through all hosts involved in transmission, which requires data to describe spatial scales and host diversity relevant to a particular context (Figure 1).

## Incorporating genomic data into schistosomiasis surveillance and control programs

Substantial challenges remain for the effective integration of genomic workflows into schistosomiasis population-based research and surveillance systems. Chief among these challenges is the generation and analysis of genomic data relevant for operational decision-making, which will require addressing interrelated issues of cost, timescales of analysis, and local infrastructure. For example, the current pattern of shipping samples from endemic regions to laboratories in high-income countries means that potentially actionable insight from genomic data is available months, if not years, after collection, which reduces the practical value of this information. Building capacity for the generation and analysis of genomic data in schistosomiasis-endemic countries is crucial to narrow this gap. Existing institutions, such as The Pathogen Genomics Institute and the African Centre of Excellence for Genomics of Infectious Disease, both affiliated with the African Centers for Disease Control, have laid important groundwork for prioritizing capacity building and in-country leadership of genomic research, as have other programs including MalariaGEN and H3Africa (Parker and Kwiatkowski, 2016). Development of standard analytical packages responsive to programmatic needs can further accelerate the integration of genomic data into programmatic decision-making. Researchers focused on schistosomiasis can also benefit from pioneering advances incorporating genomic data in Guinea Worm disease surveillance (Ribado et al., 2021) and malaria elimination programming (Dalmat et al., 2019), as well as those in rabies elimination (Brunker et al., 2020) and antimicrobial stewardship programs (Nielsen et al., 2005) operating in low- and middle-income countries.

A major impetus for writing this review was the realization that, with the declining costs and increased feasibility of WGS approaches, the costs, benefits, and trade-offs involved in the use of traditional approaches (e.g., MSATs) and reduced-representation sequencing approaches (e.g., exome sequencing, ddRADseq) have shifted. It is easy to assume that because so much more data is produced by WGS approaches that it is necessarily considerably more expensive. However, in our experience actual differences in cost per sample can be small. Considering genomes the size of schistosomes (<400 Mbp), at present, a minimal WGS study (run efficiently with 50–100 samples in parallel on a high-throughput Illumina sequencer) might be approximately 10 times the cost of an MSAT study with 10 loci. A more comprehensive MSAT study with a more statistically reliable number of loci (~100–200) would incur similar costs as the genome study but would still provide far less useful data. MSAT studies also have larger up-front costs, including design, purchase, and testing of amplification primer pairs and technical training of laboratory personnel. These cost calculations will differ for established laboratories than those getting started. Similarly, RR and exome capture approaches currently cost more (per sample as well as in terms of up-front costs) than MSATs and less than WGS. Because these approaches require less sequencing than WGS, they may only be two to five times more expensive than a small MSAT study. Up-front costs for RR approaches include experimentation with restriction enzyme pairs and digest size filtering for ddRADseq to control the fraction of the genome that is usually sequenced, while exome capture requires the design and generation of specific capture probes. Cost calculations for any laboratory will also depend on the relative availability and cost of labor, whether access to efficient sequencing is limited or involves undue delay, and the laboratory’s capability to analyze the data.

In addition to cost, additional benefits of WGS should be considered. In contrast to other types of data, WGS data is highly replicable across laboratories and technologies, giving it greater long-term comparative value. The ability to combine sequencing data across laboratories and over time expands the set of questions that can be addressed, including some that may not have been apparent when the data were first collected. However, when accuracy, replicability, long-term value, and less complex sample processing procedures are not a priority, assessing more samples with lower-resolution methods may be an appropriate choice over WGS, especially if results can be obtained more rapidly. Non-WGS methods may also be useful for preliminary or screening studies, although the extra sample handling and re-handling costs mentioned above should be considered when assessing feasibility.

We suggest that low-resolution methods in the genomic age be used within surveillance systems to detect coarse-scale genetic patterns that may represent ‘red-flags’ for further investigation. For example, they might be used for preliminary assessments related to our questions of interest: (1) evidence of hybridization between schistosome species; (2) evidence that non-human definitive hosts share a similar parasite population with humans; and (3) evidence of transmission between geographically well-separated and divergent parasite populations. Large-scale sampling to detect these phenomena may identify regions where follow-up WGS studies are warranted to investigate the biology of novel recombinants, contributions of non-human definitive hosts, and patterns of parasite import. WGS can also be done with strategic sampling across populations worldwide to detect, for example, phylogenetic histories, past geographic movement, and selected genomic variants that may have important implications for drug resistance or shifts in parasite biology relevant for control.

In the long term, standardized genomic sampling approaches (e.g., WGS) and centralized organization of publicly accessible databases will lead to compounding utility of genomic data in schistosomiasis research and surveillance. This is particularly true for questions of parasite importation, as such analyses require sampling of schistosomes from the location of interest as well as from a range of possible source populations. Like MalariaGEN, public genomic data repositories such as the Schistosomiasis Collection at the Natural History Museum (SCAN) can be leveraged for these types of analyses (Emery et al., 2012). However, available comparative datasets are limited at present, which highlights the importance of prioritizing efforts to expand the representation of schistosome sequences from a range of endemic sites. This would greatly expand the utility of genomic resources, particularly as it relates to tracking parasite spread among remaining transmission foci and for expanding genomic surveillance to inform shifts in epidemiologically relevant characteristics such as host specificity and drug resistance. The curation of such resources should be undertaken with care to ensure that endemic countries and their public health authorities benefit through data access agreements and consent procedures that adequately represent potential future use and safeguard protections for individuals against stigma and discrimination, especially as the time between data collection and phylogenetic analysis shrinks (Parker and Kwiatkowski, 2016; Johnson and Parker, 2019; Johnson and Parker, 2020).

In the near term, the incorporation of genomic data into outbreak investigations can help identify key sources of infection in residual transmission hotspots, including non-human hosts and imported infections, and help expand the geographic coverage of genomic resources. For example, China’s strategy for investigating schistosomiasis outbreaks includes surveys of human, domestic animal, and snail populations (Chen et al., 2022). Adding the collection of schistosomes to such protocols to generate genomic data can help us better identify what processes drive such outbreaks and contribute to the development of genomic data resources. An important practical challenge to such investigations is the collection of schistosomes, particularly from non-human mammalian hosts and snails (Liang et al., 2022; Kamel et al., 2021). Sensitive and efficient methods to identify and sample snail infections, which are uncommon even in high-prevalence settings, or cercariae in water would enable studies of genomic diversity of schistosomes in intermediate hosts in low-prevalence areas approaching elimination. Similarly, development of guidelines for surveying non-human mammalian hosts for schistosomiasis and collecting schistosomes will enhance the probability that infection reservoirs are identified and samples from all relevant definitive hosts are included in genomic studies.

### Conclusions

In this review, we identified two priority questions concerning why schistosomiasis reemerges and persists in different contexts: (1) how often do non-human hosts species contribute to human infection? and (2) what is the importance of locally acquired versus imported infections in driving transmission at different stages of elimination? These questions address ecological processes in schistosome transmission dynamics that can undermine ongoing treatment-based control programs. We argue that the integration of genomic data into epidemiological and ecological studies is key to answering these questions, and central to achieving and sustaining control of schistosomiasis. Genomic methods can be used to identify sources of infection, and thus help resolve social and ecological processes in ways that support decision-making about environmental interventions and targeting of non-human mammalian hosts as complements to MDA programs focused on treating people for schistosomiasis. Until recently, these questions were difficult to answer well enough to inform public health decision-making. We argue that with recent advances in genomic approaches and the development of standardized WGS datasets for comparative contexts, schistosomiasis control programs can be better tailored to local contexts with MDA programs complemented by interventions that address salient geographic and taxonomic sources of new infections.

The role of non-human animals in local transmission and imported infections can determine which additional intervention(s) may be necessary to control schistosome transmission. In our view, MDA may be sufficient if parasite populations circulate only through humans at local scales, while environmental interventions, such as snail control or WASH infrastructure, become necessary to mitigate the impact of parasite importation from elsewhere. Meanwhile, livestock treatment or wildlife surveillance are important interventions to diminish the influence of local non-human sources of infection (Figure 1). In revealing the existence and external source of human and non-human infection, whole genome approaches can help schistosomiasis researchers and practitioners maximize the impact of schistosomiasis control interventions in their jurisdictions.

## Additional files

Supplementary file 1. List of 47 studies of schistosomiasis epidemiology employing molecular methods with details on data generation method, study question, and study scale.

Supplementary file 2. References of 47 studies of schistosomiasis epidemiology employing molecular methods by data generation method, study question, and study scale.

## Appendix 1

### Search terms

We searched PubMed and Google Scholar in August 2021 for English language papers using two strings of medical subject heading (MeSH) terms related to: (1) the causative agents of human schistosomiasis (e.g., *S. haematobium, S. japonicum, S. mansoni, Schistosoma,* schistosomiasis) and (2) the use of genetic data in epidemiological and population-level studies (e.g., population genetics, molecular epidemiology, genetic epidemiology). The terms within each string were combined with the Boolean operator OR, and the strings themselves were combined with the Boolean operator AND. Additional searches using specific methodological and topical terms (e.g., microsatellite, mitochondrial DNA, hybridization, whole genome, zoonosis) were performed to supplement the results of initial searches. All search results were constrained to (1) studies involving primary data collection (i.e., excluding reviews) and (2) studies using DNA from *Schistosoma* parasites (as opposed to snails). We additionally searched the references of papers identified by our search.
